# Transient Receptor Potential Vanilloid 1 Modulates Central Inflammation in Multiple Sclerosis

**DOI:** 10.3389/fneur.2019.00030

**Published:** 2019-01-29

**Authors:** Mario Stampanoni Bassi, Antonietta Gentile, Ennio Iezzi, Sara Zagaglia, Alessandra Musella, Ilaria Simonelli, Luana Gilio, Roberto Furlan, Annamaria Finardi, Girolama A. Marfia, Livia Guadalupi, Silvia Bullitta, Georgia Mandolesi, Diego Centonze, Fabio Buttari

**Affiliations:** ^1^Unit of Neurology and Neurorehabilitation, IRCCS Neuromed, Pozzilli, Italy; ^2^Laboratory of Synaptic Immunopathology, Department of Systems Medicine, Tor Vergata University, Rome, Italy; ^3^Clinica Neurologica, Università Politecnica delle Marche, Ancona, Italy; ^4^Laboratory of Neuroimmunology and Synaptic Plasticity, University & IRCCS San Raffaele, Rome, Italy; ^5^Servizio di Statistica Medica & Information Technology, Fondazione Fatebenefratelli per la Ricerca e la Formazione Sanitaria e Sociale, Rome, Italy; ^6^Neuroimmunology Unit, Division of Neuroscience, Institute of Experimental Neurology, San Raffaele Scientific Institute, Milan, Italy

**Keywords:** TNF, endocannabinoids, neuroinflammation, microglia, IL-6, capsaicin

## Abstract

**Introduction:** Disease course of multiple sclerosis (MS) is negatively influenced by proinflammatory molecules released by activated T and B lymphocytes and local immune cells. The endovanilloid system plays different physiological functions, and preclinical data suggest that transient receptor potential vanilloid type 1 (TRPV1) could modulate neuroinflammation in this disorder.

**Methods:** The effect of TRPV1 activation on the release of two main proinflammatory cytokines, tumor necrosis factor (TNF) and interleukin (IL)-6, was explored in activated microglial cells. Furthermore, in a group of 132 MS patients, the association between the cerebrospinal fluid (CSF) levels of TNF and IL-6 and a single nucleotide polymorphisms (SNP) influencing TRPV1 protein expression and function (rs222747) was assessed.

**Results:** In *in vitro* experiments, TRPV1 stimulation by capsaicin significantly reduced TNF and IL-6 release by activated microglial cells. Moreover, the anti-inflammatory effect of TRPV1 activation was confirmed by another TRPV1 agonist, the resiniferatoxin (RTX), whose effects were significantly inhibited by the TRPV1 antagonist, 5-iodoresiniferatoxin (5-IRTX). Vice versa, BV2 pre-treatment with 5-IRTX increased the inflammatory response induced by LPS. Moreover, in MS patients, a significant association emerged between TRPV1 SNP rs222747 and CSF TNF levels. In particular, the presence of a G allele, known to result in increased TRPV1 protein expression and function, was associated to lower CSF levels of TNF.

**Conclusions:** Our results indicate that TRPV1 influences central inflammation in MS by regulating cytokine release by activated microglial cells. The modulation of the endovanilloid system may represent a useful approach to contrast neuroinflammation in MS.

## Introduction

In Multiple Sclerosis (MS), T lymphocytes and local immune cells play critical roles by releasing a number of cytokines and chemokines involved in the induction and maintenance of the inflammatory process. In addition, recent findings suggest that specific proinflammatory and anti-inflammatory molecules influence neuronal functioning, altering synaptic transmission and plasticity (1–3), and negatively influencing disease course of MS by promoting excitotoxic neuronal damage (4, 5). Among others, tumor necrosis factor (TNF) represents a proinflammatory molecule critically involved in the pathogenesis of both animal models of disease (i.e., experimental autoimmune encephalomyelitis, EAE) and of human MS (1, 6). In particular, TNF release by activated microglial cells promotes neurodegeneration since the early phases of EAE, even before the appearance of the clinical deficits. The specific role of this molecule has been also confirmed by the finding that both microglia and TNF reproduced *in vitro* the alterations observed in EAE, and the effects were prevented by blocking TNF signaling (1). In addition to TNF, different proinflammatory cytokines, have been found elevated in the cerebrospinal fluid (CSF) of MS patients (7). In the brain of MS patients, demyelinating lesions display IL-6 expression and glial cells activation (8). Moreover, this cytokine is elevated in the serum and CSF of MS patients and may negatively impact the disease course (9–11). Of note, mice lacking IL-6 gene are resistant to EAE induction (12), suggesting the pathogenic role of this cytokine in MS.

The complex relationship between inflammation and neurodegeneration involves different neurotransmitters and receptors, among them the endovanilloid system (EVS) plays a pivotal role. The transient receptor potential vanilloid type 1 (TRPV1), is a non-selective cationic channel activated by both exogenous (i.e., capsaicin, toxins) and endogenous (i.e., high temperatures, acid pH, anandamide, 2-arachidonoylglycerol) stimuli (13–15). TRPV1 modulates nociceptive responses both in the peripheral and central nervous system (CNS) (16–18), it is widely expressed in brain neurons and glial cells (19–22), and its activation modulates microglia-neuron communication (23).

In EAE, TRPV1 can modulate the inflammatory milieu regulating the release of different proinflammatory and anti-inflammatory cytokines (24). In addition, there is evidence that TRPV1 regulates the permeability of the blood-brain barrier and, due to this mechanism, it can be considered a key factor controlling the disease progression in EAE (25).

However, the factors influencing the levels of central inflammation in MS are not completely understood. In particular, it is not clear whether genetic variability of TRPV1 gene could promote neuroinflammation. Specific single-nucleotide polymorphisms (SNPs) in the TRPV1 gene could represent a suitable tool to investigate the role of this receptor channel in human pathology. Previous reports, in fact, showed that SNPs in the TRPV1 gene affect the activity of TRPV1 channel (26). In particular, the rs222747 SNP influence protein receptor expression and function (26), cortical excitability in healthy humans (27), and modulate pain in MS patients (28).

To investigate the immunomodulatory role of TRPV1 in microglia, we explored *in vitro* the effect of TRPV1 receptor stimulation on TNF and IL-6 release in activated microglial cells. In addition, to address the role of genetic variability of TRPV1 on central inflammation in MS, we examined whether TRPV1 SNP rs222747 influences TNF and IL-6 concentrations in the CSF of MS patients.

## Patients and Methods

### *In vitro* BV2 Cell Culture and TNF and IL-6 Measurement

The day before the experiment, microglial BV2 cells were plated onto 35 mm culture dishes at a density of 8 × 10^5^ in triplicates for each experimental condition in DMEM supplemented with 5% certified endotoxin free fetal bovine serum (FBS, Hyclone) and 1% penicillin/streptomycin. Next, the cells were pre-treated with TRPV1 agonists capsaicin (CAP, Tocris; 10–25 μM) and resiniferatoxin (RTX, Tocris; 1 nM) and/or antagonist 5-Iodoresiniferatoxin (5IRTX, Tocris; 1 μM) for 30 min before adding 100 ng/ml Lipopolysaccharides (LPS) or an equivalent volume of vehicle (DMSO).

Cell culture medium was collected at 6 (only for CAP treatment) and 24 h and soon centrifuged at 1,200 rpm in microfuge in order to remove any dead or detached cells. The medium was then aliquoted and stored at −80°C until use. Supernatants were assayed for TNF and IL-6 by means of Multiplex assay and the plate was read on a Luminex-200 instrument (Luminex Corp., Austin, TX). Concentrations were calculated by using a standard 5P-logistic weighted curve and expressed as pg/ml.

### MS Patients

The Ethics Committee of the University Hospital of Tor Vergata (Rome, Italy) approved the study and all patients gave a written informed consent.

One hundred and thirty two patients participated to the study. All patients were admitted to the Neurology Clinic of Tor Vergata Hospital in Rome and diagnosed as suffering from MS according to validated criteria (29). Clinical examination, brain and spine magnetic resonance imaging (MRI), blood withdrawal for SNP genotyping, and lumbar puncture (LP) for CSF collection were performed at the time of diagnosis. No patient was treated with immunoactive drugs before hospitalization, corticosteroids or disease-modifying therapies were initiated after LP.

The following demographic and clinical characteristics were collected during hospitalization: sex, age, expanded disability status score (EDSS) (30), disease duration, presence of clinical and/or radiological disease activity.

Radiological examination by 1.5 Tesla MRI included the following sequences: dual-echo proton density, fluid-attenuated inversion recovery, T1-weighted spin-echo (SE), T2-weighted fast SE, and contrast-enhanced T1-weighted SE after intravenous gadolinium (Gd) infusion (0.2 ml/kg).

### SNP Genotyping

Genotyping for TRPV1 SNPs rs222747 was performed in all patients. The MassARRAY Assay Design 3.1 software was used to design a single 20-multiplex reaction in which the SNPs of the TRPV1 gene was included. Genotyping was performed using iPLEX Gold technology (31) and MassARRAY high-throughput DNA analysis with matrix-assisted laser desorption/ionization time-of-flight mass spectrometry (Sequenom).

### CSF Sampling and Analysis

Immediately after LP, CSF samples were centrifuged and stored at −80°C. To analyze the levels of the main proinflammatory and anti-inflammatory molecules, a Bio-Plex multiplex cytokine assay (Bio-Rad Laboratories, Hercules, CA, USA) was used. The CSF levels of TNF and IL-6 were assessed. Concentrations were calculated according to a standard curve generated for the specific target and expressed as picograms/milliliter (pg/ml). All samples were analyzed in triplicate.

### Statistical Analysis

Data were presented as mean (standard deviation, sd) or, if they did not were normally distributed, as median (Interquartile Range, IQR). Kolmogorov-Smirnov test was applied to verify the normality of data distribution. Categorical variables were expressed as frequency (n) and percentage (%).

Parametric *T*-test or, when necessary, non-parametric Mann-Whitney test was applied to test difference between two categories. Chi-square test or Fisher's exact test was applied to test association between two categorical variables. In the *in vitro* experiment, differences among groups were assessed by one-way ANOVA followed by Tukey HSD. Benjamini-Hochberg (BH) adjustment was applied to control the False discovery rate in multiple comparisons. A *p* < 0.05 was considered statistically significant. All analyses were performed by statistical software SPSS.

## Results

### Activated TRPV1 in Microglia Controls TNF and IL-6 Release

**Figure 1:**
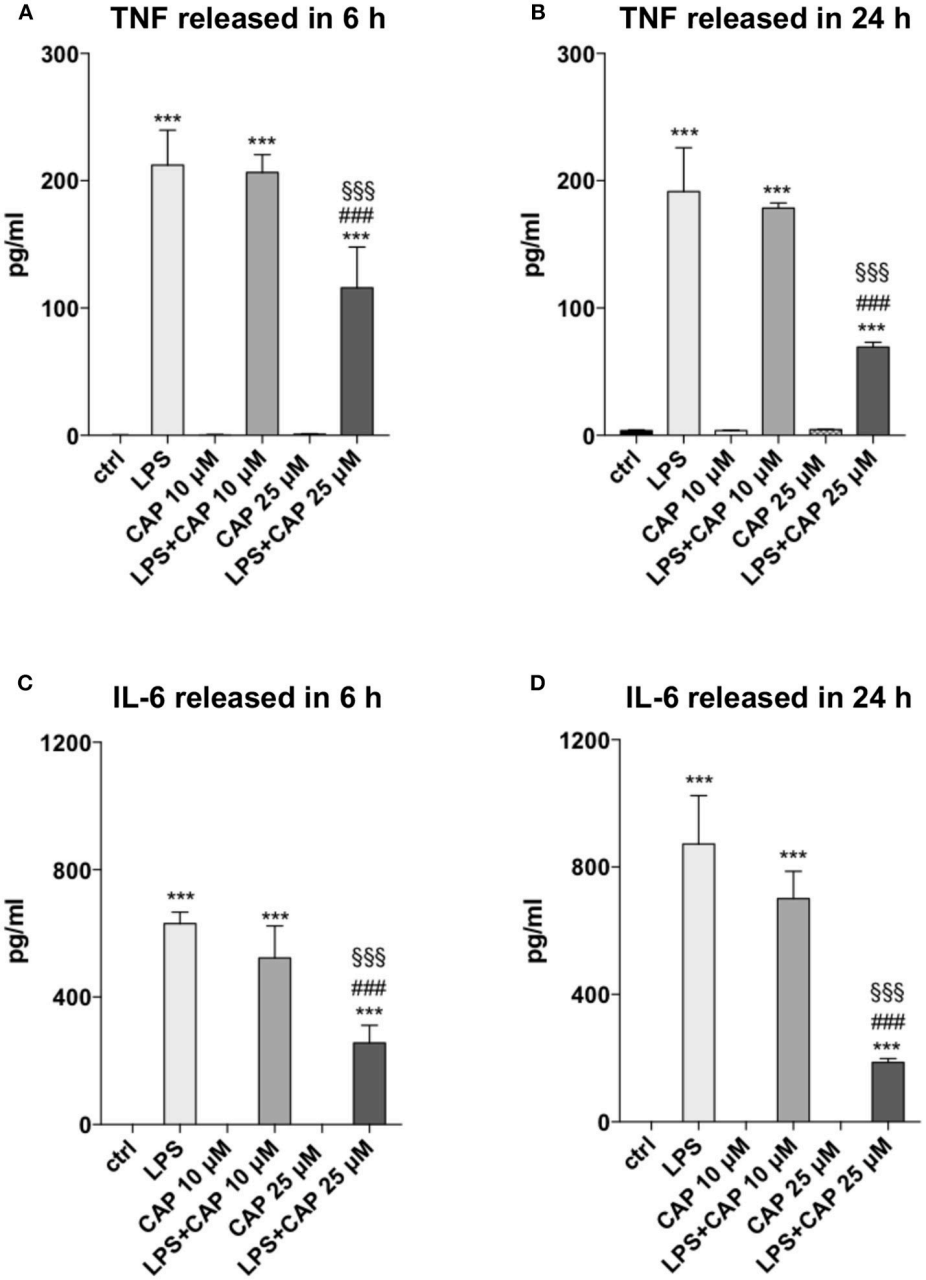
Activated TRPV1 in microglia controls TNF and IL-6 release. TRPV1 stimulation reduces TNF and IL-6 release by BV2 cells under inflammatory challenge. The levels (pg/ml) of TNF **(A,B)** and IL-6 **(C,D)** were measured in BV2 culture media harvested after 6 and 24 h of LPS treatment, respectively. One-way ANOVA *p* < 0.001, followed by Tukey HSD: ^***^p < 0.001 for comparisons of LPS, LPS+CAP 10 μM, LPS+CAP 25 μM to Ctrl; ^*###*^*p* < 0.001 for comparisons of LPS+CAP 25 μM to LPS, ^§§§^*p* < 0.001 for comparison of LPS+CAP 25 μM LPS+ CAP 10 μM.

We investigated whether microglia might be involved in controlling neuroinflammation through TRPV1, using an *in vitro* experimental paradigm of microglia activation. To assess the influence of TRPV1 stimulation on microglia response to inflammation, we pre-activated TRPV1 expressed on BV2 microglia cell line with different concentrations of the TRPV1 agonist capsaicin before adding the inflammatory stimulus, LPS. BV2 cells express functional TRPV1 (32) and are widely used as a valid alternative of primary microglia in many experimental settings (33). In our experimental conditions, TRPV1 activation did not alter *per se* the secretion of TNF and IL-6 by BV2 cells at any of the concentrations used (10 and 25 μM) and the time points considered, i.e., short-term (6 h) and long-term (24 h) (Figure 1). Notably, at both time points, pre-stimulation of TRPV1 with 25 μM capsaicin significantly reduced the LPS-induced release of TNF (for both time points One-way ANOVA *p* < 0.001, followed by Tukey HSD; *p* < 0.001 for comparisons of LPS, LPS+CAP10 μM, LPS+CAP 25 μM to Ctrl; *p* < 0.001 LPS vs. LPS+CAP 25 μM; *p* < 0.001 LPS+CAP10 μM vs. LPS+CAP 25 μM, Figures 1A,B) and IL-6 (for both time points One-way ANOVA *p* < 0.001, followed by Tukey HSD; *p* < 0.001 for comparisons of LPS, LPS+CAP10 μM, LPS+CAP 25 μM to Ctrl; *p* < 0.001 LPS vs. LPS+CAP 25 μM; *p* < 0.001 LPS+CAP10 μM vs. LPS+CAP 25 μM, Figures 1C,D) by BV2 cells. These results suggest that TRPV1 expressed on microglia can attenuate their activation during neuroinflammation.

**Figure 2:**
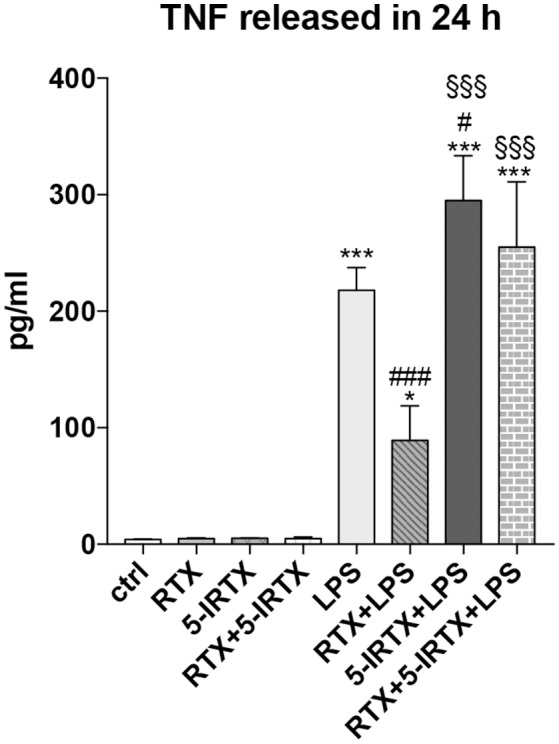
Pharmacological agonism/antagonism of TRPV1 elicits opposite responses of BV2 cells to LPS. BV2 cells were pre-treated with the TRPV1 agonist RTX (1 nM), the TRPV1 antagonist 5-IRTX (1 μM) or a combination of both before the addition of LPS. RTX alone significantly attenuated the amount of TNF released by the cells, while 5-IRTX significantly increased the TNF release induced by LPS and counteracted the anti-inflammatory effect of RTX. One-way ANOVA *p* < 0.001, followed by Tukey HSD: ^***^p < 0.001 for comparisons of LPS, 5-IRTX+LPS, RTX+5-IRTX+LPS to Ctrl, ^*^p < 0.05 for comparisons of RTX+LPS to Ctrl, ^*###*^*p* < 0.001 for comparisons of RTX+LPS to LPS, ^#^*p* < 0.05 for comparison of 5-IRTX+LPS to LPS, ^§§§^*p* < 0.001 for comparison of 5-IRTX+LPS and RTX+5-IRTX+LPS to RTX+LPS.

Studies in EAE mice have provided convincing evidence that TNF has a crucial role in MS pathogenesis (34–36). To validate the involvement of TRPV1 on the inflammatory pathway, we pre-treated BV2 cells with highly potent TRPV1 agonist, RTX (1 nM), and the potent TRPV1 antagonist, 5-IRTX (1 μM). Based on previous results, we analyzed the amount of TNF production after LPS challenge at 24 h. In line with the above data, both the molecules did not modulate the release of TNF by BV2 cells in the absence of an inflammatory challenge, while the pre-treatment of the cells with RTX confirmed the effect of inhibition of TNF release observed with CAP (Figure 2; One-way ANOVA *p* < 0.001, followed by Tukey HSD; *p* < 0.001 RTX+LPS vs. LPS; *p* < 0.05 RTX+LPS vs. Ctrl). Notably, the TRPV1 antagonist 5-IRTX increased the amount of TNF released by BV2 cells and counteracted the inhibitory action of RTX on TNF production, suggesting the critical role played by TRPV1 in the regulation of the inflammatory pathway (One-way ANOVA *p* < 0.001, followed by Tukey HSD; *p* < 0.05 for comparison of 5-IRTX+LPS to LPS; not significant the comparison between RTX+5-IRTX+LPS to LPS and 5-IRTX+LPS; *p* < 0.001 5-IRTX+LPS and RTX+5-IRTX+LPS compared to Ctrl and to RTX+LPS).

### Clinical and Demographic Characteristics of MS Patients

Clinical and radiological examination, CSF and blood sample withdrawal were performed in 132 MS patients. The clinical and demographic characteristics of patients are shown in Table 1.

**Table 1:** Demographic and clinical characteristics of MS patients.

**MS Patients**	***n***	**132**
Age, years	mean (sd)	34.8 (10.18)
Sex, Female	*n* (%)	86 (65.2%)
**MS CLASSIFICATION**		
RR	*n* (%)	122 (92.4%)
PP/SP	*n* (%)	10 (7.6%)
Disease duration, months	median (25–75 th percentiles)	8.7 (1.95–28.18)
EDSS at LP	median (25–75 th percentiles)	2 (1–2.5)
Clinical activity at LP	*n* (%)	55 (41.7%)
Radiological activity at LP	*n* (%)	48 (36.4%)

*RR, relapsing/remitting; PP, primary progressive; SP, secondary progressive; EDSS, expanded disability status scale*.

The SNP rs222747 showed no significant departure from Hardy–Weinberg equilibrium. The distribution of the SNPs rs222747 was as follows: CC (*n* = 70; 53%), CG (*n* = 54; 40.9%), GG (*n* = 8; 6.1%). For further analyses, patients were grouped as GG/GC (*n* = 62; 47%) or CC.

### TRPV1 SNP rs222747 Reduces CSF Inflammation

**Figure 3:**
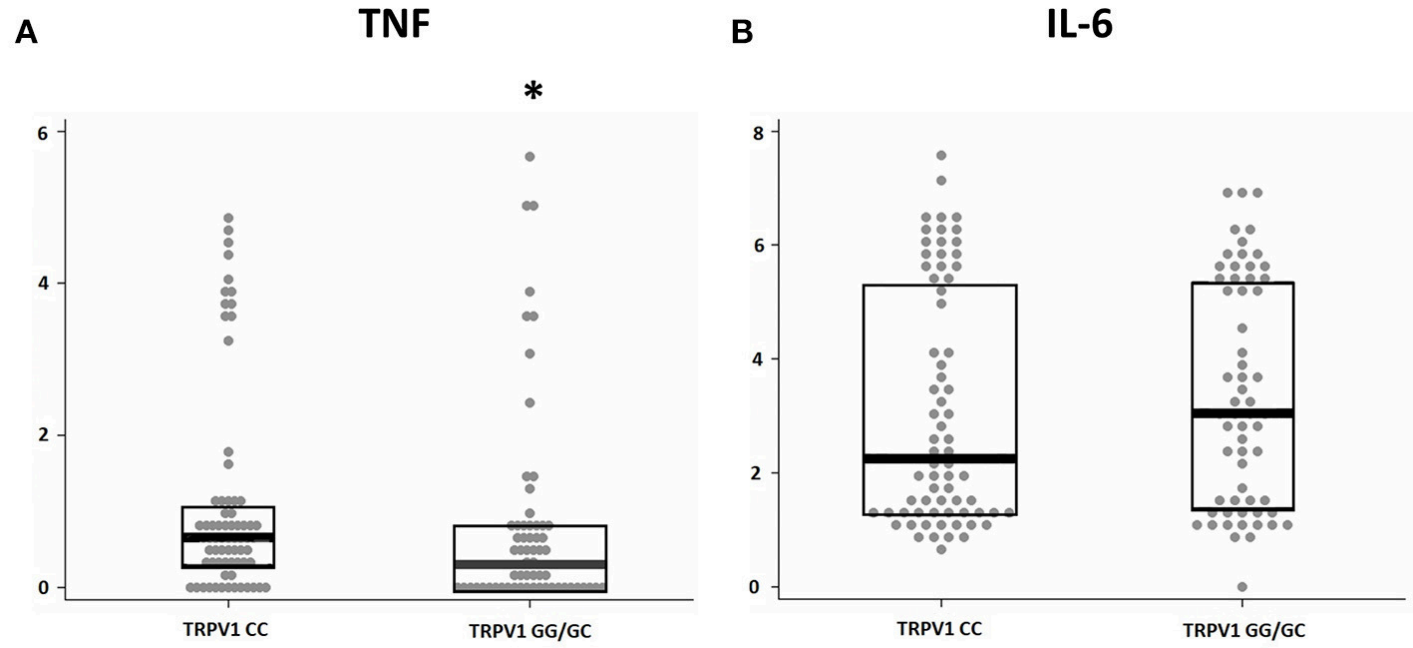
TRPV1 SNP rs222747 modulates central TNF levels in MS. TNF **(A)** and IL-6 **(B)** concentrations are shown in logarithmic scale. Higher CSF levels of TNF are observed in the TRPV1 rs222747 “CC” group. Mann–Whitney: ^*^p = 0.01.

We investigated the association between the TRPV1 SNP rs222747 genotype and the levels of TNF and IL-6 in the CSF of MS patients at the time of diagnosis. Significant associations emerged between TRPV1 rs222747 and TNF CSF concentrations. TNF values showed a skewed right distribution due to a large number of patients with very low CSF TNF levels and a relatively small proportion of patients showing very high concentrations of this molecule. The CG/GG group showed reduced CSF levels of TNF (CC *n* = 70, median = 0.94 IQR = 0.39–2.02 vs. CG/GG *n* = 62, median = 0.36 IQR = 0–1.36; *p* = 0.01, B-H adjusted *p* = 0.02) (Figure 3). No significant differences emerged in IL-6 CSF levels between the two groups (CC *n* = 70, median = 8.6, IQR = 2.75–222.69 vs. CG/GG *n* = 62, median = 20.2, IQR = 3.14–221.68; *p* = 0.496, B-H adjusted *p* = 0.496).

The clinical characteristics of the MS patients according to TRPV1 SNP rs222747 genotype (CC vs. CG/GG) are shown in Table 2. No significant differences emerged between the two groups in age, sex, disease duration, EDSS at diagnosis, presence of clinical/radiological disease activity at the time of diagnosis.

**Table 2:** Clinical characteristics of MS patients according to TRPV1 group.

		**TRPV**		***p-*value**
		**CC**	**CG + GG**	
		***n* = 70**	***n* = 62**	
Age, years	mean (sd)	34.1 (9.79)	35.7 (10.63)	0.418
Sex, Female	*n* (%)	45 (64.3%)	41 (66.1%)	0.824
**MS CLASSIFICATION**				
RR	*n* (%)	62 (88.6%)	60 (96.8%)	0.102^a^
Disease duration, months	median (25–75th percentiles)	8.9 (2.4–32.97)	8.7 (0.93–28.13)	0.515
EDSS at LP	median (25 - 75th percentiles)	2 (1–2.5)	1.5 (1–2.5)	0.150
Clinical activity at LP	*n* (%)	28 (40.0%)	27 (43.5%)	0.943
Radiological activity at LP	*n* (%)	28 (40.0%)	20 (32.3%)	0.593

a *Fisher's exact test*.

## Discussion

TRPV1 is a prominent member of the transient receptor potential (TRP) ion channel superfamily expressed in different tissues (37). In the CNS, TRPV1 has been found in neurons and resident immune cells and mediates pleiotropic functions. Although TRPV1 activation is clearly involved in the regulation of the inflammatory response (38), the role in MS central inflammation and neurodegeneration is not completely understood. In particular, the effects of TRPV1 activation may depend on the specific inflammatory milieu (39) and therefore, in MS, may have different consequences in different disease phases (40). A growing body of evidence suggests that TRPV1 expressed on microglia controls neuroinflammation. In brain resident immune cells, TRPV1 activation is associated to a wide range of functions, including cell death, phagocytosis, migration, production of cytokines and other inflammatory mediators (41–46). Intriguingly, TRPV1 activation also promotes the release of extracellular vesicles from microglia (47), likely modulating microglia-neuron communication (23).

In the present study, the association between TRPV1 functionality in microglia and TNF and IL-6 has been explored by an *in vitro* experiment designed to simulate a condition of enhanced TRPV1 activity. In particular, stimulating TRPV1 in microglial BV2 cells with 25 μM capsaicin before activation by LPS, attenuated TNF and IL-6 release from BV2. Interestingly, in human monocytes and in murine primary microglia pre-treatment with capsaicin before LPS stimulation inhibits the release of TNF, IL-1β, IL-6, prostaglandin E2 and 8-isoprostane (48). It is worth noting that both doses of the agonist used to activate TRPV1 and the stimulation timing seem crucial to regulate the response of microglia to inflammation. Indeed, 1 h treatment with 10 μM capsaicin increased the expression of IL-1β, IL-6, TNF and high mobility group box 1 (HMGB1) protein from BV2 cells (32), while in our experiment both 10 and 25 μM capsaicin did not induce changes in TNF or IL-6 release compared to control cells after neither 6 or 24 h. These findings raise the possibility that TRPV1 agonists only transiently increase the release of proinflammatory cytokines due to rapid agonist-induced receptor rundown (49). On the contrary, only 25 μM capsaicin was effective in the attenuation of cytokine release induced by LPS in BV2. Of note, the pharmacological stimulation of the TRPV1 with another TRPV1 agonist, RTX, confirmed the inhibitory effect of TRPV1 activation on the LPS-mediated inflammatory pathway in microglia cells. Moreover, the pre-treatment of BV2 cells with the TRPV1 antagonist 5-IRTX potentiated the LPS-induced activation of inflammatory route in these cells and counteracted the anti-inflammatory activity of TRPV1 stimulation, providing convincing evidence of the crucial role of TRPV1 in the modulation of microglia response to LPS challenge.

Overall, these data are in line with previous studies showing that TRPV1 activation may influence cytokine release in different inflammatory conditions. In particular, administration of a TRPV1 agonist inhibits TNF production in a rat model of arthritis (50). Moreover, in a rat model of EAE, the administration of a TRPV1 agonist reduced LPS-induced release of TNF and IL-1β, and enhanced IL-10 production (24).

Previous evidence suggests that in different inflammatory conditions TRPV1 activity may exert both proinflammatory and anti-inflammatory effects (39). For example, animal studies showed that TRPV1 activation may have anti-inflammatory activity protecting from ischemia/reperfusion injury (51), contrasting the inflammation-mediated damage in inflammatory bowel disease (52), and reducing inflammation in sepsis (53) and allergic contact dermatitis (54). Conversely, TRPV1 activation may promote proinflammatory and pro-nociceptive effects as described in animal models of peripheral neuropathic pain (55) and bone cancer pain (56), and in human osteoarthritis and rheumatoid arthritis (57).

To clarify whether TRPV1 functionality influences central inflammation also in MS, a prototypical neuroinflammatory disorder, we explored the association between two TRPV1 SNPs and the CSF levels of TNF and IL-6. TRPV1 locus is extremely polymorphic and a great number of non-synonymous SNPs have been described. In particular, SNP rs222747 is localized in the region of the Ankyrin repeat domains, and its variant is associated with increased expression and enhanced functionality of the channel (26). In particular, the presence of the G allele, compared to the wild-type CC, provides a natural model of enhanced functionality of the channel. Our results show that enhanced TRPV1 functionality in SNP rs222747 GG/GC carriers influence CSF cytokine composition in MS patients. In particular, although no clear association emerged between increased genetic functionality of TRPV1 and IL-6 CSF levels, our data in humans provided further confirmation that TRPV1 channel function regulates TNF release.

TNF is specifically involved in EAE and in MS pathogenesis. Elevated CSF levels of TNF have been in fact reported in progressive MS patients (6), and both meningeal immune cells infiltration and B cell follicles described in MS brains produce high levels of TNF (6, 58–61). Of note, inhibition of TNF signaling improves EAE (34–36).

In conclusion, we provided evidence that TRPV1 signaling regulates neuroimmune crosstalk in MS, suggesting that its pharmacological modulation could affect MS disease course and inflammatory neurodegeneration.

## Author Contributions

MS and AG: study concept and design, acquisition of data, analysis, and interpretation, first draft of manuscript; EI and FB: study concept and design, analysis, and interpretation, critical revision of the manuscript for important intellectual content; IS: statistical analyses, analysis and interpretation, critical revision of the manuscript for important intellectual content; SZ, AM, RF, AF, LiG, SB, GM, GAM, and LuG: acquisition of data, analysis and interpretation, critical revision of the manuscript for important intellectual content. DC: study concept and design, critical revision of the manuscript for important intellectual content, study supervision.

### Conflict of Interest Statement

The authors declare that the research was conducted in the absence of any commercial or financial relationships that could be construed as a potential conflict of interest.

## Abbreviations

CAP: Capsaicin
CNS: central nervous system
CSF: cerebrospinal fluid
EVS: endovanilloid system
EDSS: expanded disability status score
EAE: experimental autoimmune encephalomyelitis
FBS: fetal bovine serum
Gd: gadolinium
HMGB1: high mobility group box 1
5-IRTX: 5-iodoresiniferatoxin
IL: interleukin
IQR: interquartile range
LPS: lipopolysaccharides
LP: lumbar puncture
MRI: magnetic resonance imaging
MS: multiple sclerosis
RR: relapsing-remitting
RTX: resiniferatoxin
SNP: single nucleotide polymorphism
sd: standard deviation
SE: spin-echo
TRVP1: transient receptor potential vanilloid type 1
TNF: tumor necrosis factor.
